# A Facile and Flexible Method for On-Demand Directional Speed Tunability in the Miniaturised Lab-on-a-Disc

**DOI:** 10.1038/s41598-017-07025-x

**Published:** 2017-07-27

**Authors:** Ming K. Tan, Ariba Siddiqi, Leslie Y. Yeo

**Affiliations:** 1 Micro/Nanophysics Research Laboratory, RMIT University, Melbourne, VIC 3001 Australia; 2 School of Engineering, Monash University Malaysia, 47500 Bandar Sunway, Selangor Malaysia

## Abstract

The Miniaturised Lab-on-a-Disc (miniLOAD) platform, which utilises surface acoustic waves (SAWs) to drive the rotation of thin millimeter-scale discs on which microchannels can be fabricated and hence microfluidic operations can be performed, offers the possibility of miniaturising its larger counterpart, the Lab-on-a-CD, for true portability in point-of-care applications. A significant limitation of the original miniLOAD concept, however, is that it does not allow for flexible control over the disc rotation direction and speed without manual adjustment of the disc’s position, or the use of multiple devices to alter the SAW frequency. In this work, we demonstrate the possibility of achieving such control with the use of tapered interdigitated transducers to confine a SAW beam such that the localised acoustic streaming it generates imparts a force, through hydrodynamic shear, at a specific location on the disc. Varying the torque that arises as a consequence by altering the input frequency to the transducers then allows the rotational velocity and direction of the disc to be controlled with ease. We derive a simple predictive model to illustrate the principle by which this occurs, which we find agrees well with the experimental measurements.

## Introduction

Centrifugal microfluidic platforms, otherwise known as the Lab-on-a-CD^1–3^, have advanced considerably in the past decade with a number of technologies currently under commercial development, particularly for medical diagnostics, biosensing and drug screening applications^4–6^. A reason for the attractiveness of these platforms is their ability to circumvent a significant limitation associated with a majority of Lab-on-a-Chip systems, namely, the necessity for external pumps to drive fluid motion and particle manipulation within the microfluidic chip. By exploiting the centrifugal force acting on a CD as it spins with the aid of a rotational motor, a number of fluidic operations such as valving, metering, mixing, decanting, splitting, concentration and separation, among others, can be achieved completely within a closed system defined by channels and chambers etched in the CD^1^.

**Figure 1:**
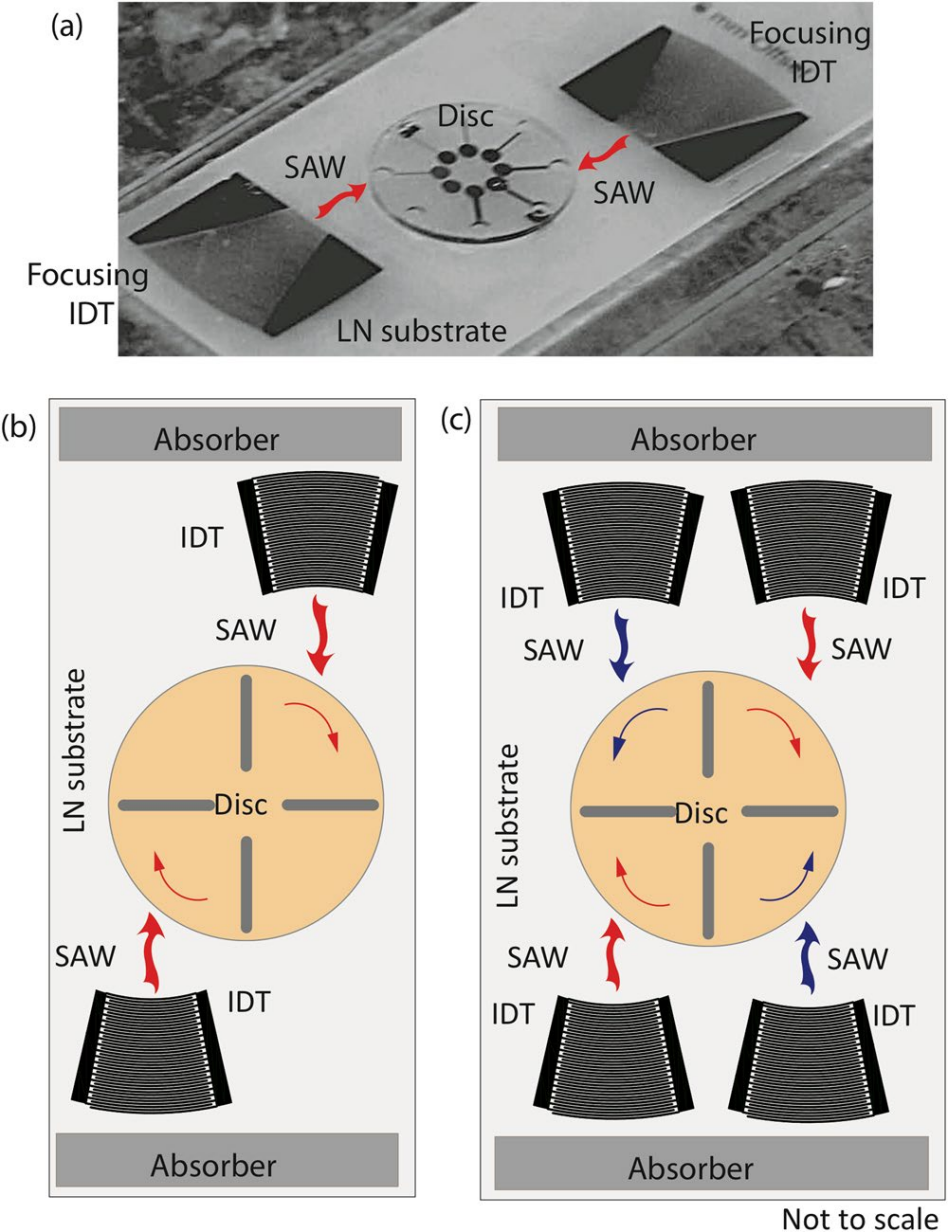
(**a**) Image (reproduced with permissions from ref. 7; © (2012) John Wiley & Sons, Inc.) and (**b**) schematic of the Miniaturised-Lab-on-a-Disc (miniLOAD) device. (**c**) A second pair of opposing offset IDTs are required in order to reverse the rotation direction of the disc. Although this is a possible solution, the range of disc dimensions that can be used for each device is extremely limited given that the IDT patterns on the substrate are fixed. Altering the disc size requires IDTs to be placed in different locations on the piezoelectric lithium niobate substrate, thus requiring a different device.

The necessity for such a motor nevertheless makes it difficult to further miniaturise the Lab-on-a-CD platform from its current benchscale dimensions to that which facilitates portable operation for true point-of-care use. In an attempt to address this, Glass *et al*. developed the Miniaturised-Lab-on-a-Disc (miniLOAD) system^7^ which exploits the introduction of asymmetry^8, 9^ to planar surface acoustic waves (SAWs)^10^—nanometer amplitude electromechanical waves that propagate on a chipscale piezoelectric substrate^8, 9, 11^—to drive azimuthal fluid rotation of a thin fluid coupling layer in order to spin a thin 10 mm disc mounted atop the fluid^7, 12^ (Fig. 1(a) and (b)). In addition, they also showed the possibility for carrying out similar fluidic operations in microchannels fabricated on the disc to that in the CD. The advantage of the miniLOAD platform, and, more generally, microfluidics driven by SAWs^13–15^, is the ability to miniaturise and integrate all its requisite components, including a battery-powered electronic driver circuit, into a lightweight (≈110 g) handheld portable device^16^.

A big drawback of the miniLOAD technology, however, lies in its inability to easily exert finer control over the disc’s rotation speed and direction that is necessary for a number of operations. This is, in part, due to the fixed pattern of the interdigitated transducer (IDT) electrodes required to drive the SAW and hence the fluid and disc rotation: the IDTs are photolithographically patterned on the piezoelectric substrate with a finger periodicity and width that relates to a preselected SAW wavelength *λ*_SAW_ and hence resonant frequency *f*_SAW_ = *c*_SAW_/*λ*_SAW_. As such, once the IDTs are patterned onto the device, it is not possible to alter this, and hence its frequency or wavelength, on the same device. This is particularly limiting if arbitrary rotational direction of the disc is desired, which can only be effected in the miniLOAD platform by patterning and triggering a second opposing pair of offset IDTs on the device whilst relaxing the power on the first IDT pair (Fig. 1(c)). The fixed position and aperture of these IDTs means that the disc size has to be fixed relative to these IDT positions; in other words, altering the disc dimension would require a new device with a separate IDT design patterned specifically to match. Similarly limiting in practice is the existing method for varying the rotational speed in the miniLOAD platform by adjusting the input power to the IDTs^7, 12^. This is because the range of input powers which can generate a rotational flow is quite narrow, ceasing below a lower threshold and giving rise to nebulisation, which is undesirable, above an upper threshold.

**Figure 2:**
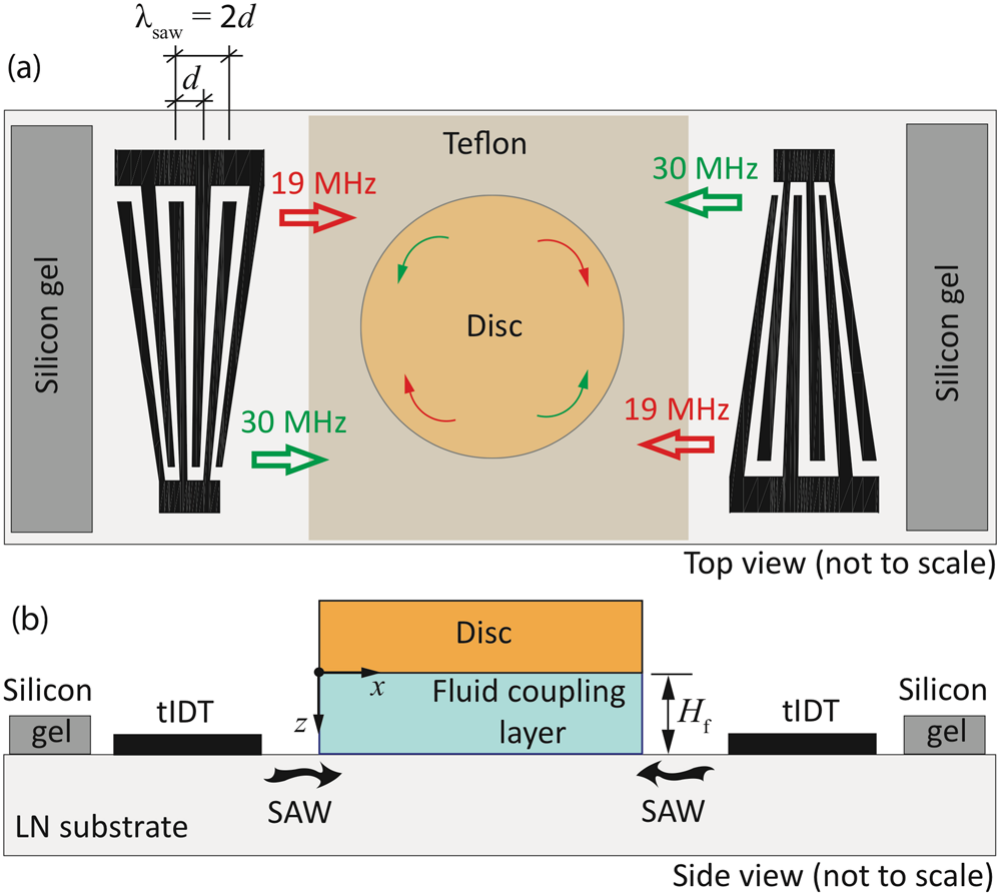
(**a**) Top, and, (**b**) side view schematics depicting the setup used in the present experiments, which comprises a pair of tapered interdigital transducers (tIDTs) patterned on the piezoelectric lithium niobate (LN) substrate. A thin disc is placed atop a fluid coupling layer which the SAW is transmitted into to drive acoustic streaming in a small strip aligned with the position where the SAW is launched, depending on the choice of the operating frequency. For the case of the tIDT design in this work, this is in the range 19–30 MHz. As illustrated, excitation of the tIDTs at a resonant frequency of 19 MHz launches a narrow width SAW at the top left and bottom right in the top view image in (**a**) so as to drive clockwise rotation of the fluid and hence the disc. Similarly, excitation of the tIDTs at 30 MHz launches the SAW at the bottom left and top right in (**a**) so as to drive counter-clockwise rotation.

In this work, we show the possibility for reconfigurability of the disc operation in terms of arbitrary and flexible variation of the disc rotation speed and direction on the same device, simply by varying the operational frequency. To do this, we make use of slanted or tapered IDTs (sIDTs or tIDTs) which possess a non-uniform periodicity between the IDT fingers such that the resonant frequency of the IDT varies along the lateral position of the tIDT. Excitation of the tIDT at a specific frequency therefore only generates a SAW with a beamwidth (or effective aperture)^17^

1 $${A}_{{\rm{eff}}}\approx (\frac{{f}_{{\rm{SAW}}}}{{f}_{{\rm{U}},{\rm{SAW}}}-{f}_{{\rm{L}},{\rm{SAW}}}})\frac{{A}_{{\rm{t}}}}{N}$$

at a local position on the tIDT that matches this resonant condition (*f*_L,SAW_ ≤ *f*_SAW_ ≤ *f*_U,SAW_, in which *f*_L,SAW_ is the lower SAW frequency, *f*_U,SAW_ the upper frequency, *A*_*t*_ the total tIDT aperture and *N* the number of finger pairs). By judicious choice of the operating frequency, it is then possible to launch a confined SAW to produce fluid motion in a narrow strip at very specific locations on the device (Fig. 2). This strategy has been successfully used in a number of other microfluidic manipulations such as drop translation^18^, mixing^19, 20^, breakup and splitting^21, 22^, liquid/particle steering and deflection^23–28^, liquid/particle trapping^29, 30^, nebulisation^31^, rotation on superstrates^32, 33^, and the generation of spatial temperature gradients^34^. Here, the specific position of the SAW and hence actuation of fluid motion in a narrow strip beneath the disc through the choice of the operating frequency imparts a force at a specific lateral distance from the disc’s centre, and hence, in doing so, gives rise to a torque on the disc (Fig. 2), which we exploit to control its rotational speed and direction. This capability is independent of the disc’s dimension since this force can be imparted at any lateral location along the disc, as long as its diameter is narrower than the IDT aperture.

More specifically, the devices used in the current work consist of a 128°-rotated *Y*-cut *X*-propagating single-crystal lithium niobate (LN) piezoelectric substrate (Roditi Ltd, London, UK) on which an opposing pair of tIDTs (Fig. 2), fabricated using standard sputter deposition and photolithographic processes documented elsewhere^7^, are patterned. The tIDTs have a broad frequency band *f*_SAW_ = *c*_SAW_/2*d*, wherein *d* is the finger spacing (see Fig. 2(a)); in this work, *f*_SAW_ ranges between *f*_L,SAW_ = 19 MHz and *f*_U,SAW_ = 30 MHz across the lateral width of the tIDTs. To generate the SAW at a given location, a sinusoidal electric signal at a specific frequency within this range is applied to both tIDTs using a function generator (SML-01, Rohde & Schwarz, North Ryde, NSW, Australia) and a high frequency amplifier (10W1000C, Amplifier Research, Souderton, PA, USA) via a pair of SMA cables and custom-made electrical contact probes. Exciting the tIDT pair at the same frequency thus launches mutually opposing acoustic radiation beams into the coupling layer symmetric about its center to drive acoustic streaming in a narrow strip within the fluid corresponding to the SAW beam width, leading to azimuthal fluid motion in the entire fluid coupling layer. The direction of the disc rotation can thus be reversed simply by switching the frequency of the SAW such that the acoustic radiation enters from the opposing diagonal ends of the fluid layer (see, for example, Fig. 2 wherein excitation at *f*_SAW_ ≈ 19 MHz drives clockwise rotation whereas excitation at *f*_SAW_ ≈ 30 MHz gives rise to anticlockwise rotation). To prevent the spreading of the fluid due to acoustowetting effects^35^ which leads to precession of the disc, a thin hydrophobic layer was coated in the area between the fluid coupling layer and the tIDTs. To do this, the tIDT pair and a 10 mm diameter circular region (corresponding to the dimension of the disc and hence the fluid coupling layer beneath it) was first masked with tape, after which we spin coated a thin layer of Teflon^®^ AF (polytetrafluoroethylene; The Chemours Company, Wilmington, DE, USA) onto the surface, followed by baking at 80 °C for 1 hr to form a strong hydrophobic surface that would not be removed by the propagating SAWs; we note that across the range of applied electrical powers used, no damage of the Teflon^®^ coating was observed over repeated experiments with the same device. 10 mm diameter discs were fabricated using a hole punch through a 100 *μ*m thick Mylar^®^ (polyethylene terephthalate; DuPont Teijin Films, Hopewell, VA, USA) sheet. In the experiment, the disc was balanced on top of a fixed volume ($\sim 100\,\mu \ell$) water coupling layer; we estimate, from mass conservation, that the thickness of this fluid coupling layer *H*_f_ is approximately 1.3 mm. Disc rotation was recorded using a high speed camera (iSpeed, Olympus, Essex, UK) attached to a long working distance lens (Infinivar CFM-2/S, Infinity, Boulder, CO, USA); the angular velocity of the disc was then estimated using these recorded images.

**Figure 3:**
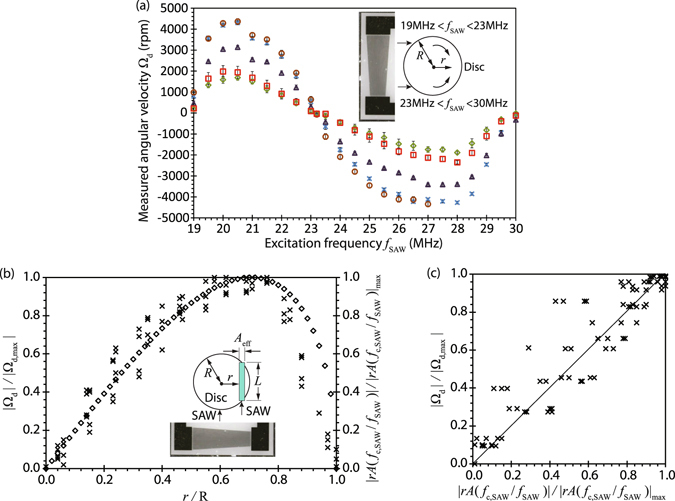
(**a**) Experimental measurements showing the relationship between the rotational speed of the disc Ω_d_ and the applied frequency *f*_SAW_ to the tIDTs for different electrical input powers: (○) −1 dBm, (×) −2 dBm, (Δ) −4 dBm, (□) −5 dBm and (◇) −6 dBm. The inset shows the approximate position of the disc relative to the tIDT, and indicates the frequency range *f*_SAW_ which drives clockwise (19–23 MHz) and anti-clockwise (23–30 MHz) rotation. The error bars indicate the standard deviation of the data taken across triplicate measurements. (**b**) Experimentally measured (×) disc rotational velocity Ω_d_ (normalised by its maximum value Ω_d,max_) as a function of the radial position where the SAW is launched from the tIDT with respect to the disc’s centre *r* (normalised by the disc radius *R*), compared to the prediction afforded by the simple theoretical model (◇). The inset shows a schematic illustrating the position of the disc with respect to the tIDT and the strip under the disc with chord length *L* and effective aperture *A*_*eff*_ corresponding to the area *A* where the SAW is launched to give rise to acoustic streaming under the disc. (**c**) Recast of the plot in (**a**) showing the linear relationship between the disc rotational velocity Ω_d_ and the theoretical prediction *rA*(*f*_c,SAW_/*f*_SAW_).

Figure 3(a) shows the possibility for easily varying the disc’s rotational direction and speed Ω_d_ over a range from 0 up to 4400 rpm simply by adjusting excitation frequency *f*_SAW_ applied to the tIDTs together with the input power. It can be seen that the disc rotates anti-clockwise between 19 and 23 MHz, and clockwise between 23 and 30 MHz. This is because the applied frequencies below 23 MHz tends to generate the SAW closer toward the left-side of the disc (looking from the tIDT towards the disc; see Fig. 3(a), inset) where the finger periodicity of the tIDT is larger, whereas applied frequencies above 23 MHz tends to generates the SAW closer toward the right-side of the disc. At approximately 23 MHz, the SAW is aligned towards the centre of the disc and hence there is no net torque on the disc; as a consequence, the disc has no net motion, i.e., it has zero rotational speed. We note the slight asymmetry about this frequency, as opposed to symmetry about the tIDT centre frequency (*f*_U,SAW_ − *f*_L,SAW_)/2 = 24.5 MHz. This could arise due to the difficulty in exact symmetric placement of the disc around the centre location. Alternatively, it is also possible that the asymmetry arises due to the slight nonlinearity in the way the frequency varies with the position along the tIDT^36^.

No observable differences in the location of the SAW that is generated and hence the rotation speed of the disc were recorded for the input powers (and hence substrate vibration displacement amplitudes) applied over the range which allowed for stable disc operation. The frequency range is also too narrow to cause significant changes in the acoustic streaming behaviour in the liquid as a consequence of its influence on the attenuation length of the sound wave in the liquid^37, 38^. We note though the asymmetry in the frequency range, i.e., 19–23 MHz and 23–30 MHz, arises due to the relative position of the disc along the lateral length of the tIDT and the slight nonlinearity in the way the frequency varies with the position along the tIDT^36^; as such, the results in Fig. 3(a) should be interpreted as representative rather than definitive in nature, changing depending on where the disc is placed relative to the tIDT. We also note that the maximum rotational speed is attained when the SAW is excited at a lateral location along the tIDT that coinciding with the mid-point of the disc’s radius, i.e., *r* = *R*/2, corresponding to *f*_SAW_ ≈ 20.5 and 27.5 MHz.

We developed a simple model to demonstrate how the torque imparted on the disc *T*_avg_ arises, which we assume to primarily be due to the average shear stress exerted on it by the SAW-induced acoustic streaming and azimuthal flow in the fluid coupling layer

2 $${\tau }_{{\rm{avg}}}\approx \mu {(\frac{\partial {u}_{{\rm{dc}},x}}{\partial z})}_{z=0},$$

where *u*_dc,*x*_ is the acoustic streaming velocity in the SAW propagation direction, and *z* is the coordinate orthogonal to the substrate surface in the vertical direction, with *z* = 0 corresponding to the disc surface. As such,

3 $${T}_{{\rm{avg}}}\approx {\tau }_{{\rm{avg}}}A,$$

wherein *A* = *LA*_eff_ is the area on the underside of the disc defined by a strip of length *L* and the effective aperture *A*_eff_ that corresponds to the area where the narrow beamwidth of the SAW leaks its energy into the fluid coupling layer to drive the streaming, as shown in the inset of Fig. 3(b); we note that *L* can be estimated graphically from the chord length of the disc at the specific lateral position where the SAW is launched, which, in turn, depends on the frequency at which the tIDT is excited. Given *H*_f_ ≈ 10^−3^ m is much smaller than the viscous attenuation length^39, 40^ ${\alpha }_{{\rm{f}}}^{-1}=2{\rho }_{{\rm{f}}}{c}_{{\rm{f}}}^{3}/[b{(2\pi {f}_{{\rm{SAW}}})}^{2}]\approx {10}^{-2}-{10}^{-1}$ m, wherein *ρ*_f_ is the fluid density, *c*_f_ its sound speed, and *b* = 4 *μ*/3 + *μ*_B_, with *μ* and *μ*_B_ being the shear and bulk viscosities of the liquid, respectively, the sound waves that are generated in the fluid coupling layer due to the leakage of the SAW energy into it do not attenuate appreciably through the thickness of the fluid coupling layer and are therefore subject to multiple reflections between the underside of the disc and the substrate. Nevertheless, it is reasonable to assume, justified by the good agreement between the model predictions and the experimental results that will be seen below, that the radiation pressure on the disc as the sound waves impinge and reflect off the disc does not contribute significantly to its rotation. This is, in part, due to the near vertical angle at which the SAW leaks into the fluid and hence the angle at which the sound wave impinges on the disc—defined by the Rayleigh angle *θ*_R_ = sin^−1^ *c*_SAW_/*c*_f_ ≈ 22° to the vertical—such that the horizontal component of the radiation force acting on the disc is small. Moreover, given an acoustic impedance *r*_*a*_ ≈ *ρ*_f_ *c*_f_, which, ﻿for water *r*_a,w_1.5 × 10^6^ kg/m^2^ s and Mylar^®^ *r*_a,d_ = 3.6 × 10^6^ kg/m^2^ s, the power transmission coefficient^40^ ${T}_{{\rm{\Pi }}}=4{r}_{{\rm{rt}}}{\theta }_{{\rm{rt}}}/{[{r}_{{\rm{rt}}}+{\theta }_{{\rm{rt}}}]}^{2}\approx \mathrm{78 \% }$, where *r*_rt_ = *r*_a,d_/r_a,f_ and *θ*_rt_ = cos *θ*_t_/cos*θ*_i_, indicates that only around 22% of the incident acoustic radiation is reflected back into the fluid coupling layer; note that *θ*_t_ and *θ*_i_ are the angle of transmission and incidence of the acoustic wave, respectively.

Given that the rotational velocity of the disc Ω_d_ varies linearly with the average torque acting on it, it follows from Eqs (2) and (3) that

4 $${{\rm{\Omega }}}_{{\rm{d}}}\sim {T}_{{\rm{avg}}}\sim rA\mu {(\frac{\partial {u}_{{\rm{dc}},x}}{\partial z})}_{z=0},$$

wherein *r* is the radial distance between the disc’s centre and the position of the strip where the SAW and hence the acoustic streaming beneath the disc is excited. Since ${H}_{{\rm{f}}}\ll {\alpha }_{{\rm{f}}}^{-1}$, the acoustic streaming velocity in the fluid region immediately beneath the disc surface *z* → 0 scales as^41^

5 $${u}_{{\rm{dc}},x}\sim \frac{{\rho }_{{\rm{f}}}{\delta }_{v}{u}_{x}{u}_{z}}{2\mu }\exp (-z/{\delta }_{v}),$$

where *u*_*x*_ and *u*_*z*_ are the instantaneous acoustic particle velocities in the *x*- and *z*-directions, respectively, and *δ*_v_ ≡ [*μ*/(*πρ*_f_ *f*_SAW_)]^1/2^ is the boundary layer thickness^10^. Assuming that the power transmitted from the SAW substrate into the fluid coupling layer *P*_a_ ~ *u* ^2^ *f*_SAW_ remains constant irrespective of the frequency, we then note that the instantaneous acoustic particle velocity $u\sim \mathrm{1/}{f}_{{\rm{SAW}}}^{\mathrm{1/2}}$. Choosing the tIDT frequency that coincides with exciting a SAW and hence the acoustic streaming along the disc centre where *r* = 0 as a reference frequency, i.e., *u* ~ (*f*_c,SAW_/*f*_SAW_)^1/2^, we then obtain from Eqs (4) and (5) a scaling relationship for the disc’s rotational velocity:

6 $${{\rm{\Omega }}}_{{\rm{d}}}\sim rA(\frac{{f}_{{\rm{c}},{\rm{S}}{\rm{A}}{\rm{W}}}}{{f}_{{\rm{S}}{\rm{A}}{\rm{W}}}}),$$

which, from Fig. 3(b) and 3(c), can be seen to be in good agreement with the velocities measured experimentally, when normalised against the maximum rotational velocity, therefore inspiring confidence in the accuracy of the model, despite its simplicity.

In summary, we have demonstrated a way to flexibly control both the rotational direction and speed of a disc in the miniLOAD device by varying the applied frequency using tIDTs. Not only is the method simple, it also circumvents the need for manual intervention or the use of different devices to alter the frequency when a disc of a different size is to be used. We find good agreement between the theoretical results we obtain for the rotational velocity with the experimental measurements, thus validating the predictive capability of our simple model.

### Data availability

All data generated or analysed during this study are included in this published article.
